# Microbiological and Nutritional Analysis of Lettuce Crops Grown on the International Space Station

**DOI:** 10.3389/fpls.2020.00199

**Published:** 2020-03-06

**Authors:** Christina L. M. Khodadad, Mary E. Hummerick, LaShelle E. Spencer, Anirudha R. Dixit, Jeffrey T. Richards, Matthew W. Romeyn, Trent M. Smith, Raymond M. Wheeler, Gioia D. Massa

**Affiliations:** ^1^AECOM Management Services, Inc., LASSO, Kennedy Space Center, Merritt Island, FL, United States; ^2^NASA UB, Kennedy Space Center, Merritt Island, FL, United States

**Keywords:** lettuce, microgravity, ISS, phyllosphere, rhizosphere, space, microbes

## Abstract

The ability to grow safe, fresh food to supplement packaged foods of astronauts in space has been an important goal for NASA. Food crops grown in space experience different environmental conditions than plants grown on Earth (e.g., reduced gravity, elevated radiation levels). To study the effects of space conditions, red romaine lettuce, *Lactuca sativa* cv ‘Outredgeous,’ plants were grown in Veggie plant growth chambers on the International Space Station (ISS) and compared with ground-grown plants. Multiple plantings were grown on ISS and harvested using either a single, final harvest, or sequential harvests in which several mature leaves were removed from the plants at weekly intervals. Ground controls were grown simultaneously with a 24–72 h delay using ISS environmental data. Food safety of the plants was determined by heterotrophic plate counts for bacteria and fungi, as well as isolate identification using samples taken from the leaves and roots. Molecular characterization was conducted using Next Generation Sequencing (NGS) to provide taxonomic composition and phylogenetic structure of the community. Leaves were also analyzed for elemental composition, as well as levels of phenolics, anthocyanins, and Oxygen Radical Absorbance Capacity (ORAC). Comparison of flight and ground tissues showed some differences in total counts for bacteria and yeast/molds (2.14 – 4.86 log_10_ CFU/g), while screening for select human pathogens yielded negative results. Bacterial and fungal isolate identification and community characterization indicated variation in the diversity of genera between leaf and root tissue with diversity being higher in root tissue, and included differences in the dominant genera. The only difference between ground and flight experiments was seen in the third experiment, VEG-03A, with significant differences in the genera from leaf tissue. Flight and ground tissue showed differences in Fe, K, Na, P, S, and Zn content and total phenolic levels, but no differences in anthocyanin and ORAC levels. This study indicated that leafy vegetable crops can produce safe, edible, fresh food to supplement to the astronauts’ diet, and provide baseline data for continual operation of the Veggie plant growth units on ISS.

## Introduction

Crop production in space may be a necessary and desirable component of future exploration systems (MacElroy et al., 1992; Kliss et al., 2000). Fresh produce can be grown *in situ* to supplement a stored, packaged diet, and crops may provide beneficial nutrients as well as dietary variety. Veggie is a small plant growth chamber designed and built by Orbital Technologies Corporation (Now Sierra Nevada Corp., Madison, WI, United States) to grow vegetable crops in space (Morrow et al., 2005; Morrow and Remiker, 2009).

**FIGURE 1:**
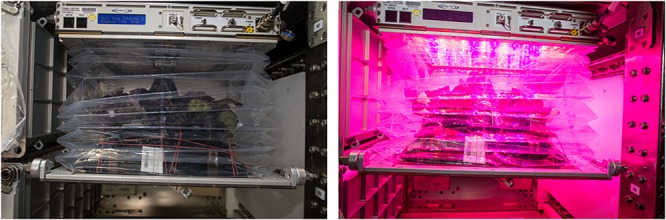
Veggie payload containing a crop of mature red romaine lettuce in Veggie pillows aboard the ISS with the light panel off **(left)** and red light panel on **(right)**. Bellows are in the up position.

The first Veggie plant growth chamber was launched to the International Space Station (ISS) in April, 2014 along with eighteen plant (rooting) pillows for the VEG-01 experiment. Veggie is a simple plant growth facility that uses LED lights and fans to circulate ISS air through the plant growth volume. A transparent, extensible bellows attached to the light unit via magnets contains the growing plants and any debris, and directs air flow from the bottom of the canopy to the top of the growth volume (Figure 1). Screens remove large particles from the cabin air before it passes through the plant growth chamber, however, no level of filtration is present, so plants growing in Veggie are exposed to any microbial or chemical constituents present in the ISS environment. More details about the Veggie facility can be found in Massa et al. (2016). For the VEG-01 and VEG-03 demonstration tests, plants were grown from seeds in plant pillows. Plant pillows are small growing bags that interact with a root mat water reservoir on the Veggie baseplate. Pillows contain a calcined clay substrate mixed with controlled release fertilizer and wicks for seed attachment. Surface sanitized seeds are glued into these plant pillow wicks, and pillows are packaged for flight under sterile air. Further details of seed and pillow preparation are provided by Massa et al. (2017b).

A major focus of our research in the past several years has been to identify crop candidates with high potential for dietary supplementation, given the constraints associated with the current space diet. Ongoing testing has indicated that some essential human nutrients are either deficient in the processed and pre-packaged space foods (e.g., potassium, Vitamin K) or may degrade to inadequate levels over the times required for long duration missions (e.g., Vitamin B1, Vitamin C) (Cooper, 2013). Several of these nutrients may be supplemented through fresh salad crops and will provide antioxidants and phytochemicals in a natural, whole-food form. Previous studies with salad crops have focused on combinations of environmental conditions (e.g., light, temperature, CO_2_ concentration) needed to optimize plant growth for space settings (Richards et al., 2004, 2006). While the macro nutritional value of crops was often evaluated in productivity studies (calories, protein, fats, and carbohydrates), micro nutritional data are limited (McKeehen et al., 1996; Wheeler et al., 1996, 1997). In order to utilize “pick-and-eat” produce such as the lettuce crops grown in VEG-01 and 03 as a supplemental food for space, nutritional assessment is essential. In this study we compare the nutritional quality of red romaine lettuce (*Lactuca sativa*, cv. Outredgeous) grown on ISS to ground controls grown under the same ISS environmental conditions including relative humidity, CO_2_ concentration and temperature. Within a Veggie chamber, conditions such as humidity and temperature can vary from ISS conditions outside the chamber, for example, the temperature and humidity inside the Veggie tend to be slightly elevated compared to the open cabin air. These differences may have an impact on the elemental, nutritional and microbiological components of the plant. Increased temperatures have been shown to decrease macronutrient content in red leaf lettuce while increasing flavonoids and phenolics (Sublett et al., 2018). Studies have shown that increased humidity initiates a positive response in biomass yield in many agricultural crops while concomitantly decreasing transpiration rate altering the uptake of water and some nutrients (Tibbitts, 1979; Gislerød and Mortensen, 1990).

Microbiological testing is an integral part of any food safety program to verify quality, production and proper handling processes but few studies exist for space grown plants (Hummerick et al., 2010). We conducted microbiological testing of Veggie-grown produce to understand potential risks to astronauts as consumers. Good agricultural practices and procedures are in place to minimize microbial contamination of crops grown in Veggie, and microbial testing is one tool to verify the effectiveness of these processes (Hummerick et al., 2011). Plant-growth conditions, the surrounding environment, mineral nutrition, and plant species can all influence the microbial populations living on plant surfaces (Lindow and Brandl, 2003). A variety of microorganisms can be found on fresh produce, most as part of the normal flora of the plant with no adverse effect on humans if consumed (Heaton and Jones, 2008; Leff and Fierer, 2013; Oyinlola et al., 2017).

Microbiological analysis can also provide insight on the microbial ecology (population density and types of bacteria and fungi) of Veggie-grown plants. The likelihood of contamination and growth of potential human pathogens on plants grown in Veggie is low since sterilization/sanitization of seeds, plant growth medium, irrigation water, and hardware can be controlled. However, if a contamination event were to occur, the risks to the crew of exposure to food borne pathogens could be increased considering the association of immune dysregulation to spaceflight (Borchers et al., 2002). Heterotrophic bacterial counts and the presence of Enterobacteriaceae that serve as an indicator of contamination of processed foods, irrigation water, and food processing surfaces are frequently present in raw foods like fresh produce making those tests irrelevant as risk indicators for fruits and vegetables (Heaton and Jones, 2008; Leff and Fierer, 2013). The microbial load on crops intended for consumption by astronauts presumably should fall within an acceptable range of microbiological standards set for food. Currently, however, there are no standards in place for microbial levels in fresh produce grown in space. The closest related NASA standards are for non-thermostabilized food items including any of the freeze dried foods consumed in orbit, e.g., shrimp cocktail, chocolate candy, granola bars. For example, the current limit for aerobic bacteria on a non-thermostabilized food item is <2 × 10^4^ colony-forming units (CFU) per gram on one sample or <1 × 10^4^ on two out of five samples (Perchonok et al., 2012) however, these standards apply to prepackaged food sent from Earth. For fresh produce grown on ISS, the microbiological acceptability is assessed on a case-by-case basis relying on microbiological analysis of crops grown in ground studies analogous to Veggie grown crops. Microbiological analysis has been performed previously on edible plants grown in the Russian Lada chamber on ISS (Hummerick et al., 2010, 2011). Mizuna, barley and radish from Lada were stored at −80°C after harvest until analysis was performed at Kennedy Space Center, Merritt Island, FL, United States. A range of microbial densities as indicated by aerobic bacterial and fungal plate counts was found depending on plant type and location. The four samples of mizuna, the only leafy green tested, were grown in different Lada root modules at different times and counts ranged from 3.1 × 10^3^ – 8.7 × 10^5^. When compared to field grown or market produce of similar types these numbers are well within the expected range of microbial densities or even lower (Holvoet et al., 2015; Wood et al., 2015; Fröhling et al., 2018; Hummerick et al., unpublished data).

However, not all microorganisms can be cultured in the laboratory and alternate methods of community characterization are needed (Byrd et al., 1991; Schaule et al., 1993; Kalmbach et al., 1997). Genomic DNA analysis using whole genome or amplicon sequencing provides the capability of identifying 98–99% of the microbes present in a complex, heterogeneous microbial community. With the current advances in technology, the Illumina MiSeq provided a platform for capturing the community structure of the leaf and root material returned from the ISS and for the ground control samples. Identifying the community makeup is the first step in understanding the complex structure and interactions between mutualistic or symbiotic partners in plant growth systems.

The purpose of this study was to determine the effects of ISS and Veggie environmental conditions on the microbiological and nutritional quality of Veggie grown lettuce intended for crew consumption.

## Materials and Methods

### Plant Growth and Harvest

The Veggie production system on ISS is a small plant growth chamber designed and built by ORBITEC (Madison, WI, United States) to grow crops in space (Morrow et al., 2005; Morrow and Remiker, 2009). The Veggie production system, equipped with LED lighting and a passive watering system, launched to the ISS in 2014 aboard Space X’s third Commercial Resupply (CRS-3) mission (Figure 1). Red romaine lettuce *Lactuca sativa* cv. ‘Outredgeous’ was grown in Veggie rooting pillows as described by Massa et al. (2017a). Briefly, plants were grown in Veggie pillows containing solid, porous, arcillite (calcined clay) substrate in two different blends, either 100% sized to 600 μ–1 mm or a 1:1 ratio of that size to 1–2 mm (Turface Proleague, Profile Products, LLC) and controlled release fertilizer (Nutricote 18-6-8, type 180, Florikan, Sarasota, FL, United States). Procedures for detailed substrate and pillow preparation are outlined in Massa et al. (2017b). Two surface sanitized seeds were attached with guar in each plant pillow between paired germination wicks. All plant rooting pillows with attached seeds were kept dry in heat sealed, gas-impermeable Tedlar bags until test initiation on the ISS. Seeds for three separate, independent plantings (biological replicates) were germinated and grown for 33–56 days on ISS and ground controls were run in controlled environment chambers under ISS environmental conditions with a 24 h delay for VEG-01A and B and a 72 h delay for VEG-03A. Each experiment began with six rooting pillows. Germinated seeds were thinned to one plant per pillow at day 7. Each independent planting was grown at different times aboard ISS with parallel ground experiments for comparison (Table 1).

**TABLE 1:** Initiation and harvest schedule of red romaine lettuce crop in Veggie aboard ISS.

**Experiment**	**Initiation date**	**Harvest date**
VEG-01A	5/8/2014	6/10/2014 (Day 33)
VEG-OIB	7/8/2015	8/10/2015 (Day 33)
VEG-03A	10/25/2016	2nd 12/09/2016
		4th 12/28/2016

*Ground controls were harvested 1 day later for VEG-01 and 3 days later for VEG-03.*

VEG-01A was grown from May 8 to June 10, 2014; VEG-01B was grown from July 8 to August 10, 2015, and VEG-03A was grown from October 25 to December 28, 2016. VEG-01A and B were harvested after 33 days of growth using sanitized scissors to remove the leaves (Table 1). VEG-03A leaves were harvested sequentially starting at 33 days followed by 3 weekly harvests. Harvested leaves from each plant were wrapped in foil and placed into a −80°C freezer. The first, third, and half of the fourth harvests were consumed by the crew. Only the 2nd harvest and the remaining leaves (approximately half of the total leaves) of the final harvest from VEG-03A were stored for sample return. After completion of the final harvest for all three plantings, two rooting pillows from each independent experiment were removed from Veggie hardware and placed into bags and frozen at −80°C. Ground control samples were processed and stored the same as flight samples. The Veggie baseplate was positioned 30.5–31.75 cm below the LED light array to provide approximately 200 μmol⋅m^2^⋅s^–1^of photosynthetically active radiation at the surface of the pillows in the center of the Veggie unit at a ratio of 12 red (630 nm): 3 Blue (455 nm): 1 green (530 nm). A photoperiod was set to 16 h light/8 h dark. Environmental conditions from the three experiments are summarized in Supplementary Table S1. Both the VEG-01A and part of the VEG-03A experiment on ISS had data collected inside the Veggie chamber using a temperature and relative humidity data logger.

### Sample Processing

After return to Kennedy Space Center, Merritt Island, FL, United States samples from all three experiments were maintained in a −80°C freezer until analysis. Plant samples were removed from the freezer and processed immediately while pillows were thawed at 4°C overnight. Leaf biomass was divided for microbiological and chemical analysis. The samples intended for chemical analysis were subsequently frozen at −80°C, freeze dried, then ground up using an IKA Tekmar A10 Analytical Grinding Mill. Roots were removed from each pillow and divided for microbiological and molecular analysis.

### Microbiological Analysis

Leaf and pillow root samples were placed into sterile 50 ml centrifuge tubes with sterile phosphate buffered saline (PBS) with glass beads, weighed then shaken vigorously for 2 min. Sample extracts were diluted into PBS and appropriate dilutions were plated in duplicate onto trypticase soy agar (TSA) and inhibitory mold agar (IMA). Plates were incubated at 30°C for 48 h for TSA and 72–120 h for IMA before enumeration of colonies. Individual colony phenotypes were selected for each sample and streaked for isolation. Isolated bacterial colonies were identified using the Micro Id System (Biolog, Hayward, CA, United States) or MicroSEQ 16S rDNA sequencing kit for bacteria (Thermo Fisher Scientific, Waltham, MA, United States). Fungal colonies were identified using the MicroSEQ D2 LSU rDNA kit for fungi (Thermo Fisher Scientific, Waltham, MA, United States). Sequencing was completed on the ABI 3130 Genetic Analyzer (Thermo Fisher Scientific, Waltham, MA, United States). Bacterial and fungal DNA sequences were identified using MicroSEQ ID Software V2.0 (Bacterial Library, 2009; Fungal Library, 2011, Applied Biosystems, Life Technologies, Foster City, CA, United States) and/or NCBI Basic Local Alignment Search Tool (BLAST).

For microbial food safety screening, sample extracts were plated onto *E. coli*/coliform and Staph Express Petrifilm (3M, St. Paul, MN, United States) according to manufacturer’s instructions. Petrifilms were incubated at 35°C for 24 h and colonies positive for *E. coli* and *S. aureus* were enumerated. Coliform colonies, if present, were re-isolated and further identified using Biolog GEN III plates. Screening for Salmonella was done by inoculating buffered peptone water (BPW) with 1 ml of sample extract followed by incubation at 35°C for 24 h. For selective enrichment, 1 ml of the incubated BPW was transferred into 5 ml of Rappaport-Vassiliadis (RV) broth or Tetrathionate broth (Thermo Fisher Scientific, Waltham, MA, United States) and incubated for 24 h at 35°C. Broths were then streaked onto selective media for *Salmonella* and incubated at 35°C for 24–48 h. These methods were adapted from the FDA bacteriological analytical manual (FDA U.S. Food and Drug Administration, 2018).

### Microbial DNA Isolation, 16S and ITS PCR, and Sequencing

DNA was isolated from sample material processed for microbiological analysis described above. Each sample was centrifuged at 13,000 rpm to pellet microbial cells, suspended in RNA*later* and stored at −80°C until processing for DNA isolation was completed. DNA was extracted from plant leaf and root material of the Veggie pillows using the Microbial Cell DNA Isolation Kit (Qiagen, Inc, Carlsbad, CA, United States) then quantified using the Qubit 2.0 double stranded DNA assay (Invitrogen, Grand Island, NY, United States). The variable 4 region of the 16S ribosomal RNA gene (rRNA) was selected as a phylogenetic marker to identify microorganisms taxonomically to the species level. A dual indexing system for multiplexing was adapted for these communities and polymerase chain reaction (PCR) was optimized for reagent concentrations and volume. Each community was labeled with two barcoded primers for identification at completion of sequencing. Amplicons were created in triplicate using 1 ng of template DNA, and final reagent concentrations of 1X buffer, 200 μM dNTP’s, 25 mM MgCl_2_, and 300 nM each barcoded 16S rRNA gene primer for bacterial identification or barcoded ITS primers for fungal identification (Bokulich and Mills, 2013; Kozich et al., 2013). The PCR cycling conditions were 95°C for 5 min to denature the Taq polymerase enzyme, followed by 30 cycles of 95°C for 1 min, 58°C for annealing and 72°C for 2 min for extension. A final 10 min extension of 72°C completed the PCR run on a Bio-Rad C-1000 thermocycler. After PCR all amplicons were purified using the Min-Elute System (Qiagen, Carlsbad, CA, United States) and quantified with the Qubit 2.0 high sensitivity ds DNA assay (Invitrogen, Grand Island, NY, United States). The purified amplicons were then pooled in an equimolar concentration to create the library following Illumina protocols. The sample library was combined with a 10% Phi-X control library to create diversity and sequenced on the Illumina MiSeq using a V2 500-cycle kit (Illumina, Inc, San Diego, CA, United States).

### Sequence Analysis

All 16S and ITS FASTQ sequences were obtained using the Illumina MiSeq Control Software and default settings for demultiplexing all samples. Sequence analyses for 16S sequences were completed using RDP GreenGenes (DeSantis et al., 2006) to obtain the taxonomic reference. ITS FASTQ results were analyzed with the UNITE database (Kõljalg et al., 2013) to analyze the reference sequences and for taxonomic assignment. The Shannon Index was calculated for alpha diversity for each sample with MiSeq control Software (Illumina, Inc. San Diego, CA, United States) while the Bray–Curtis (Anderson, 2001) statistics methods calculated beta diversity between samples.

### Total Anthocyanins

Approximately 100 mg of each dry sample powder was placed in an ASE350 cell of the Dionex Automated Solvent Extraction System (Dionex Corporation, Sunnyvale, CA, United States) and extracted with a solvent mixture of methanol:water:acetic acid (MWA) 85:14.5:0.5 (V/V/V) (methanol and acetic acid; Sigma-Aldrich, St. Louis, MO, United States). Parameters for the ASE 350 were: 100°C with 5 min static time; 70% flush; 90 s purge; 1 cycle; and 1500 psi. Extracts were analyzed immediately after extraction for total anthocyanin content by reading their absorbance at 530 and 650 nm (Beckman DU 700). A standard curve was created using Cyandin-3-Glycoside (Sigma-Aldrich, St. Louis, MO, United States).

### ORAC and Total Phenolic Content

A separate extract was prepared for the determination, firstly, of the antioxidant capacity using the hydrophilic Oxygen Radical Adsorption Capacity (ORAC-FL) assay and secondly, the total phenolics in the sample. Twenty five milligrams (25 mg) of the freeze dried sample was placed in an ASE 350 cell and extracted with acetone:water:acetic acid (AWA) at the ratio of 70%:29.5%:0.5%. Parameters for the ASE 350 were: 80°C with 5 min static time; 60% flush; 60 s purge; 1 cycle; and 1500 psi. Aliquots of 0.2 ml were subsequently reacted with Folin-Ciocalteu phenol reagent (Sigma Aldrich, St. Louis, MO, United States) for the determination of total phenolics by the modified Folin-Ciocalteu assay (Prior et al., 2005). A series of known concentrations of gallic acid (Sigma Aldrich, St. Louis, MO, United States) was prepared and reacted with the same reagent to create a calibration curve that was in turn used to determine the concentration in the sample extracts. Consequently, the total phenolics were expressed as gallic acid (GA) equivalents. A diluted (1/50) AWA extract was subjected to the ORAC-FL assay as described by Wu et al. (2004) and Prior et al. (2005). Specifically, dilutions of the AWA extracted samples were prepared in a 75 mM phosphate buffer solution (pH 7.0). Aliquots of 20 μL of the diluted sample were placed (in triplicate) in a 96 well transparent flat bottom microplate (Thermo Fisher Scientific, Waltham, MA, United States), along with appropriately diluted Trolox [(±)-6-Hydroxy-2,5,7,8-tetramethylchromane- 2-carboxylic acid; Sigma-Aldrich, St Louis, MO, United States] standards (in triplicate), under subdued light. A “forward-then-reverse” pattern was used to place samples in the microplates, and edge wells were not used for standards or samples due to possible plate effects. Samples were analyzed on a BioTek Synergy Hybrid plate reader (BioTek, Winooski, VT, United States) which automatically added 200 μL of diluted fluorescein (Sigma-Aldrich, St. Louis, MO, United States) into each well, followed by 20 μL of the peroxyl radical generator (AAPH [2′2′azobis (2-amidinopropane)] Sigma-Aldrich, St. Louis, MO, United States). The assay was monitored every few minutes for 2 h, at 37°C. The ORAC-FL value was calculated from the area under the decay curve, and was reported as μmol Trolox equivalents (TE)/g dry weight.

### Elemental Analysis

Dried and ground plant material (0.2–0.5 g) was digested in an open vessel system using a graphite-heating block (Mod Block, PN 4370-010007, CPI International). The plant material was digested at 95°C using a modification of the Environmental Protection Agency, (EPA) Method 3050B, as described below. A 5 ml aliquot of 70% nitric acid (70% Trace Metal Grade, Fisher Scientific, Suwanee, GA, United States) was added to the samples and then boiled for approximately 2 h (or until sample was completely clear). After cooling 2.5 ml of 30% hydrogen peroxide (Fisher Scientific, Suwannee, GA, United States) was applied. When the peroxide reaction ceased, samples were reheated for an additional 50 m in covered vials. Samples were cooled overnight, diluted to 50 ml with ultra-pure DI water and then passed through a 25 mm 0.45 μm syringe filter (GE Whatman, Pittsburgh, PA, United States).

Samples were analyzed via Inductively Coupled Optical Emission Spectrometry. (iCAP 7000 Series, Thermo Fisher Scientific, Waltham, MA, United States) A multi-element standard (Environmental Express) was diluted to the same acid concentration as the samples and quantification was done by external calibration.

### Statistical Analysis

Data from microbiological counts (log transformed) and chemistry values were compared using a one-way ANOVA followed by Tukey’s multiple comparisons test using GraphPad Prism version 8.0.0 for Windows (GraphPad Software, San Diego, CA, United States). Alpha diversity was determined using the Shannon Diversity Index and was calculated by Illumina RDP Control Software (Illumina, San Diego, CA, United States). Beta diversity of community sequencing was determined with Bray–Curtis dissimilarity (Anderson, 2001) using a one-way ANOVA (QIIME 2.0) (Bolyen et al., 2018). A *t*-test (Microsoft Excel) was done to compare differences in the percent phyla identified.

## Results

### Microbial Counts on Leaves and Roots

**FIGURE 2:**
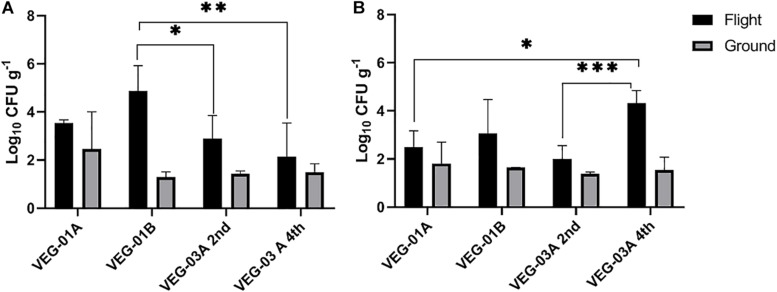
Mean bacterial **(A)** and fungal **(B)** counts on Outredgeous leaves from three Veggie experiments. Bacterial counts are CFU on TSA per gram of frozen tissue and fungal counts are CFU from IMA plates. Error bars represent standard error of the mean. Horizontal lines indicate significance between flight samples. *P < 0.05, ^∗∗^*P* < 0.01, ^∗∗∗^*P* < 0.001. Significant differences were determined using an ANOVA with Tukey’s post test to compare groups.

**FIGURE 3:**
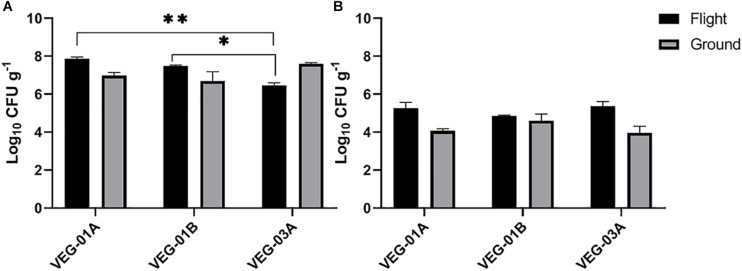
Mean bacterial **(A)** and fungal **(B)** counts on Outredgeous roots from three Veggie grow outs. Bacterial counts are CFU on TSA per gram of frozen tissue and fungal counts are CFU on IMA plates. Error bars represent standard error of the mean. Horizontal lines indicate significance between flight samples. ^∗^*P* < 0.05, ^∗∗^*P* < 0.01. Significant differences were determined using an ANOVA with Tukey’s post test to compare groups.

VEG-01A was the first time the Veggie facility had been used for plant growth on ISS (Massa et al., 2017a, b) so presumably it would have been the least likely of the three plantings described in this study to harbor microbial contamination. Aerobic plate counts for the leaves harvested from VEG-01B flight plants were significantly higher than the counts from both harvests of VEG-03A flight and the ground controls (*P* < 0.05) (Figure 2). With the exception of VEG-01B, the flight and ground control aerobic plate counts on leaves were not significantly different. The bacterial counts on ground control samples from VEG-01B were significantly lower than the flight leaves by orders of magnitude (*P* < 0.0001) (Figure 2). Previous studies (Hummerick et al., 2010, 2012) have shown an increase in microbial counts with a repeated harvest or “cut and come again” protocol as was done in VEG-03A. An increase between the 2nd and 4th harvests was not seen in bacterial counts on the leaves, however, fungal counts in the flight leaves were significantly higher in the 4th harvest when compared to the 2nd harvest (*P* = 0.0002) (Figure 3).

The bacterial counts on lettuce leaves grown on ISS in VEG-01A, B and both harvests from VEG-03A were relatively low on average, 3.53, 4.86, 2.89, and 2.14 log_10_ CFU/g respectively (Figure 2), when compared to farm or market produce counts of similar type (Valentin-Bon and Jacobson, 2008; Rastogi et al., 2012; Jackson et al., 2013; Holvoet et al., 2015; Wood et al., 2015; Hagenmair and Baker, 2016; Oyinlola et al., 2017; Fröhling et al., 2018; Zhang et al., 2018). Fungal counts on Veggie grown lettuce ranging from 2.3 to 4.3 log_10_ CFU/g were within the range of yeast and mold counts on lettuce reported in the literature (Figure 3) (Oliveira et al., 2010).

Bacterial counts from the roots in the VEG-03A grow out were significantly lower than the other two grow outs and lower than the corresponding ground control (Figure 3) although these counts were in a range of 10^6^-10^8^ per gram which is typical of lettuce root tissue (Riser et al., 1984; Adesina et al., 2009).

Screening for potential food borne pathogens, *E. coli, Salmonella sp.*, and *S. aureus* on the leaf tissue yielded negative results. These screening results were corroborated by NGS data.

### Cultivated Bacterial and Fungal Isolate Identification

Cultivation of individual bacterial colony phenotypes on TSA from VEG-01 and VEG-03 samples yielded 14 genera identified from the leaf samples (Table 2) and 19 from the root samples (Table 3). It is important to note that culture based isolation utilized in this study is limited to the selection of the mostly dominant colony phenotypes that grew under aerobic conditions on one type of general growth media. A majority of the bacterial taxa isolated from the Veggie grown leaves are known endophytic and/or epiphytic phyllosphere commensals or symbionts including *Arthrobacter* (Scheublin and Leveau, 2013), *Methylobacterium* (Peredo and Simmons, 2018), *Sphingomonas* (Kim et al., 1998), *Pantoea* (Whipps et al., 2008), *Burkholderia* (Eberl and Vandamme, 2016), and *Curtobacterium* (Chase et al., 2016). *Paenibacillus* is ubiquitous in soil and is associated with plants predominately as a rhizosphere bacterium exhibiting plant growth promoting characteristics (Grady et al., 2016). A few of the leaf isolates are known to be transients on the surface of the leaf and are not typically part of the normal phyllosphere microbial community including *Bacillus* and *Staphylococcus* (Maduell et al., 2008; Dees et al., 2015). Human associated bacteria of the genus *Staphylococcus* were found on the leaves of both flight and ground samples from VEG-03A. These are not natural inhabitants of the plant phyllosphere, however certain strains can be pathogenic to humans and cause food borne illness under optimal growth conditions (Kadariya et al., 2014).

**TABLE 2:** Bacterial isolates recovered and identified from leaf tissue from two separate rooting pillows from three independent experiments grown aboard ISS with parallel ground studies for comparison.

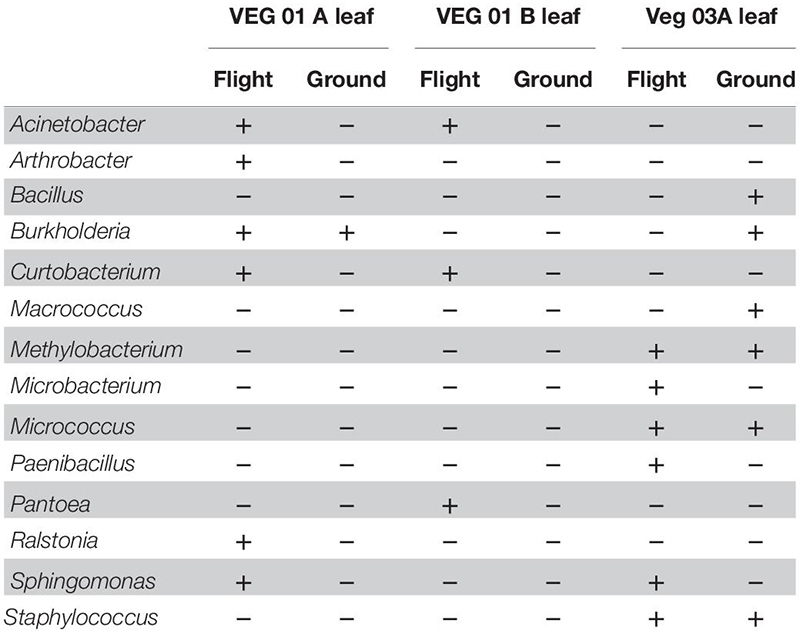

*Identification was completed with Biolog and/or MicroSEQ protocols. A ± indicates the presence or absence of each genus in flight or ground samples.*

**TABLE 3:** Bacterial isolates recovered and identified from root tissue from two separate rooting pillows from three independent experiments grown aboard ISS with parallel ground studies for comparison.

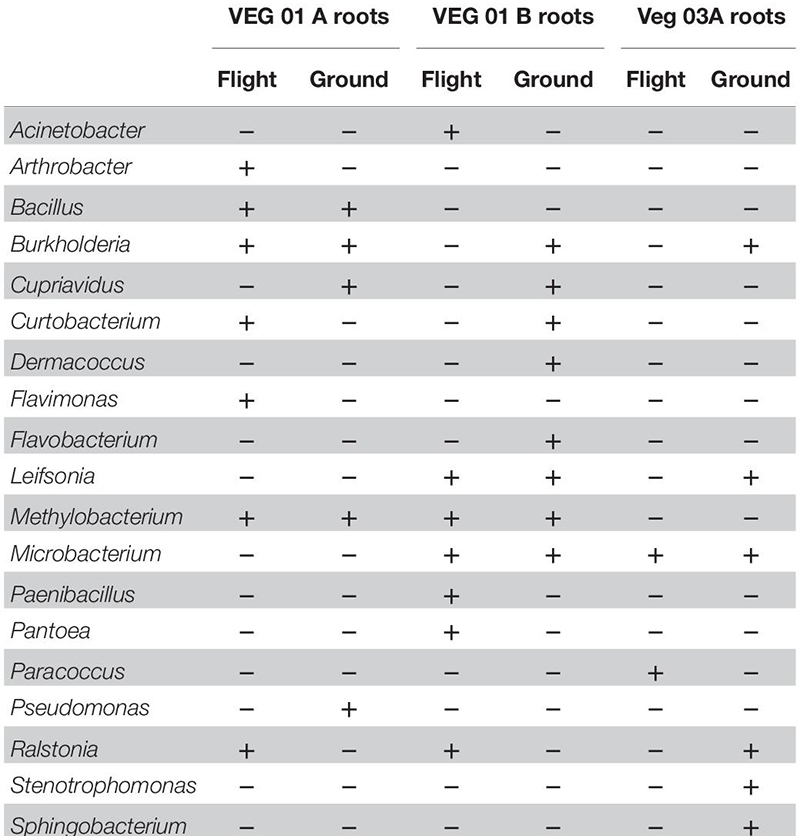

*Identification was completed with Biolog and/or MicroSEQ protocols. A ± indicates the presence or absence of each genus in flight or ground samples.*

The bacterial genera isolated from the roots (Table 3) are predominately associated with soil and plant phyllosphere and rhizosphere. Ten of the 19 root isolates were also recovered from the leaf samples excluding the four genera *Macrococcus, Micrococcus*, *Staphylococcus*, and *Sphingomonas*.

Nine genera of fungi were isolated and identified from leaf tissue (Table 4), while six were recovered from root (Table 5). All the fungal genera present in the roots were also present on the leaves with the exception of *Alternaria*. The fungi *Aspergillus, Penicillium*, and *Alternaria* are ubiquitous, saprophytic fungi often isolated from soil and the environment including ISS surface and air samples (Royer et al., 2004; Venkateswaran et al., 2017). Verma et al. (2011) found many of these genera of filamentous fungi as endophytes in the Indian Lily plant root. Among the fungi, the basidiomycete, *Rhodotorula* was the most common and in fact was isolated from every Veggie sample with the exception of the VEG-03 leaf tissue.

**TABLE 4:** Fungal isolates recovered and identified from leaf tissue from two separate rooting pillows from three independent experiments grown aboard ISS with parallel ground studies for comparison.

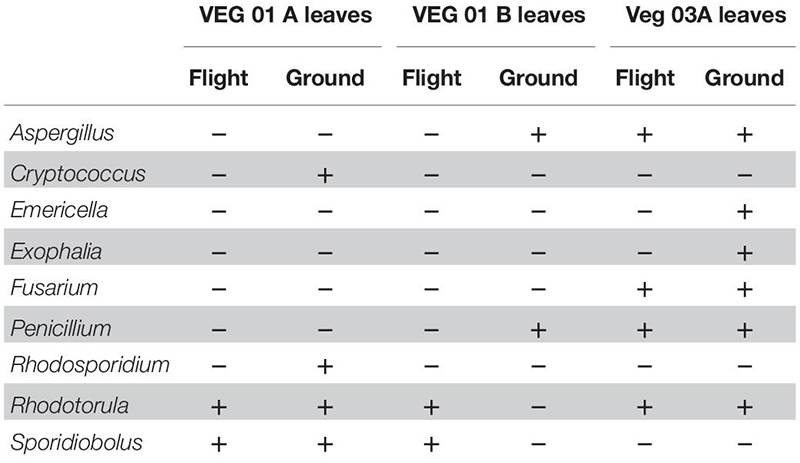

*Identification was completed with Biolog and/or MicroSEQ protocols. A ± indicates the presence or absence of each genus in flight or ground samples.*

**TABLE 5:** Fungal isolates recovered and identified from root tissue from two separate rooting pillows from three independent experiments grown aboard ISS with parallel ground studies for comparison.

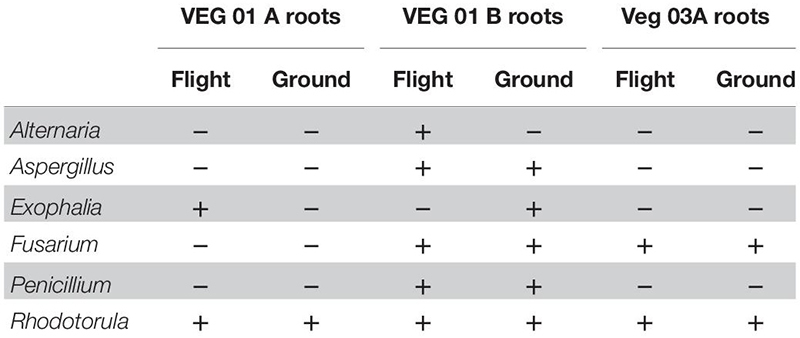

*Identification was completed with Biolog and/or MicroSEQ protocols. A ± indicates the presence or absence of each genus in flight or ground samples.*

### Community Sequencing

Sampling and sequencing of the flight and ground Veggie grown plants were completed on a minimum of two root pillows and up to five leaf samples per grow out. Sequencing profiles for each of the Veggie plantings were used to calculate and investigate the Shannon Species Diversity Index (Alpha Diversity) (Table 6) and establish relative diversity within the bacterial communities in leaf and root and ground and flight samples. Leaf diversity was lower than root in each of the three lettuce crops and there was no significant difference between flight and ground samples (Table 6). Although diversity was similar in all roots or leaves, there appeared to be variation in the dominant genera.

**TABLE 6:** Average Shannon Diversity Indices for three, independent Veggie experiments VEG-01A, VEG-01B, and VEG-03A, on the International Space Station and parallel ground studies.

**Sample ID**	**Average shannon species diversity**		
	**VEG-01A**	**VEG-01B**	**VEG-03A**
**Flight - Root**	2.46 ± 0.002	2.66 ± 0.3	1.96 ± 0.21
**Flight - Leaf**	0.517 ± 0.02	0.8 ± 0.2	0.351 ± 0.1
**Ground - Root**	2.291	2.16 ± 0.17	2.17 ± 0.002
**Ground - Leaf**	0.462 ± 0.02	0.5 ± 0.0	0.36 ± 0.09

*Alpha diversity index was generated from next generation sequencing results run on the Illumina MiSeq. Plus/minus values are standard deviation determined from three separate sample replicates within each experiment.*

Alpha diversity in leaf tissue of the three Veggie experiments showed a significant difference between ground and flight leaf samples of VEG-03A only (*P* = 0.01). VEG-01A and B flight samples were not significantly different from the respective ground samples. There was also no significant difference in Beta diversity between VEG-01A, VEG-01B, and VEG-03A experiments. Bray Curtis dissimilarity calculations comparing all three experiments were not significantly different between any leaf or root samples of the three tests (*P* > 0.05).

**FIGURE 4:**
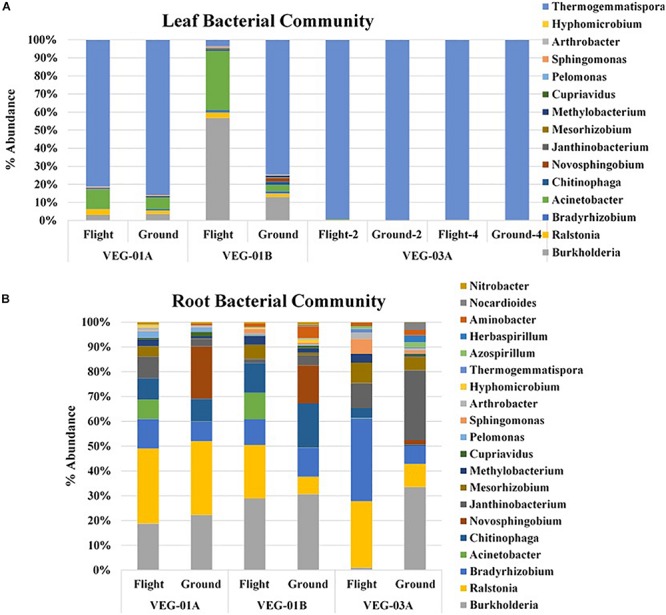
Bacterial community for lettuce leaf **(A)** and root **(B)** tissue for VEG-01A, VEG-01B, and VEG-03A experiments. Flight experiments were grown on the ISS while ground controls were grown in Environmental Growth Chambers at Kennedy Space Center, Merritt Island, FL, United States. Bacteria are the top genera obtained from next generation sequencing on the Illumina MiSeq.

A majority of the bacterial community sequencing at the phyla level were assigned to phyla Proteobacteria, Bacteroidetes, Actinobacteria, and Chloroflexi. Comparison at the phyla taxonomic level indicated no significant difference between ground and flight root communities nor in the leaf communities. The dominant phyla for all plants, both ground and flight, was Proteobacteria. VEG-03A flight roots had the lowest percent of the Proteobacteria reads at 59% while the corresponding flight leaf sample was 76% of the total bacterial communities. All other plants from VEG-01A and B ranged from 80 to 96% Proteobacterial reads. However, this disparity was not significantly different. A closer look at the genera contained in the Veggie leaf and root samples showed that *Burkholderia, Ralstonia*, and *Janthinobacterium* dominated the Proteobacteria phyla (Figure 4). Other Proteobacteria present in the samples were *Azospirillum*, and *Herbaspirillum*, which were detected in higher abundance in VEG-03A as well as *Bradyrhizobium* and *Mesorhizobium*, all of which may play a role in nitrogen fixation (Okubo et al., 2012). The dominant genera representing the Chloroflexi was the gram positive, thermophilic, *Thermogemmatispora*, a soil microbe (Komaki et al., 2016). These microbes were also transported to the leaf but were detected in much lower abundances (percentage), particularly in the flight and ground samples in VEG-03A. *Thermogemmatispora* was also detected in higher percentages than other microbes but was less than in VEG-03A. The dominant microbe representing the Bacteroidetes, which took second to the Proteobacteria in numerous samples was *Chitinophaga. Chitinophaga* was higher in abundance of reads in flight sample roots with only VEG-01B showing an elevated abundance in the ground samples (Figure 4).

**FIGURE 5:**
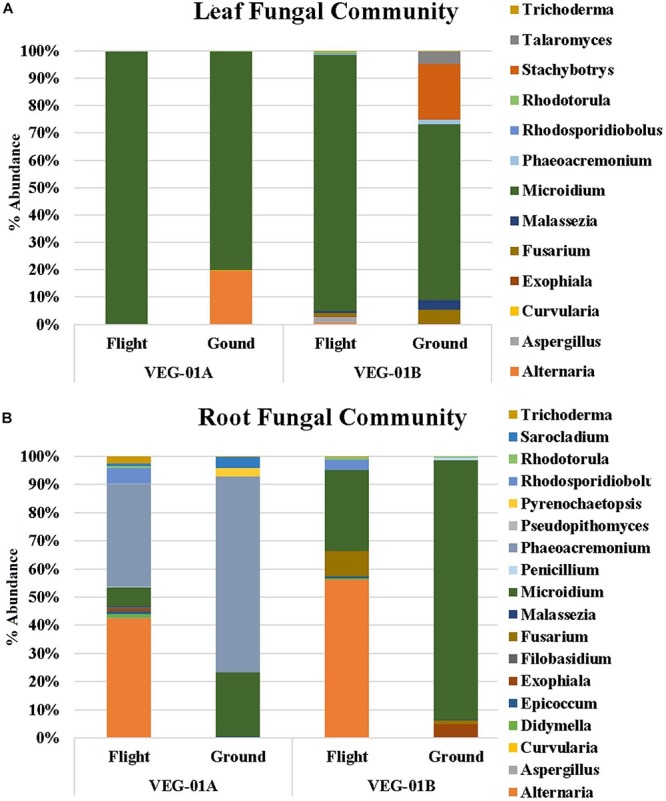
Fungal community for lettuce leaf **(A)** and root **(B)** tissue for VEG-01A and VEG-01B experiments. Flight experiments were grown on the ISS while ground controls were grown in Environmental Growth Chambers at Kennedy Space Center, Merritt Island, FL, United States. Fungi are the top genera obtained from next generation sequencing on the Illumina MiSeq.

Fungal communities from VEG-01A and VEG-01B were surveyed and it was determined that the Ascomycota phyla dominated both flight and ground communities as well as leaf and root communities (92–99%). Few Basidiomycota were detectable. VEG-01A flight roots presented the highest representation at 7% with VEG-01B flight root community containing 6%. All other communities contained 2% or less of total fungi detected (Figure 5).

Fungal diversity in the root samples for both flight and ground was significantly higher than leaf – an expected outcome (*P* < 0.05). Among the dominating genera in flight and ground leaf samples was *Microidium*, an obligate biotroph (Braun and Cook, 2012; Meeboon and Takamatsu, 2017). Leaf samples also housed *Alternaria*, *Fusarium* (VEG-01B only), and *Malassezia*, a yeast like fungi. Root samples from all three growth tests had increased levels of *Alternaria, Phaeoacremonium*, and *Rhodosporidiobolus* (Figure 5). *Alternaria* was found in all samples with the exception of VEG-01A leaf tissue while *Phaeoacremonium* was present in increased abundance in VEG-01A root and in reduced numbers in VEG-01A leaf tissue (Figure 5).

Comparison of culturable and non-culturable microbes in all samples provided corroborating evidence in that all culturable microbes were identified in the sequencing data. However, sequencing data revealed many additional genera present, increasing the species diversity.

### Elemental Analysis

Leaf tissue from flight and ground experiments were analyzed for changes in elemental composition (Table 7). There was no significant differences between ground and flight samples within each experiment, however when comparing among all three experiments, Fe and K content were significantly lower in VEG-03A samples compared to VEG-01A and VEG-01B (Fe, *P* ≤ 0.001; K, *P* ≤ 0.0001). On the other hand, Na content was found to be significantly higher in VEG-01B compared to VEG-01A or VEG-03A (*P* ≤ 0.0001). Phosphate (P) measured among all three experiments showed that VEG-01B had higher P contents than VEG-01A and VEG-03A (*P* ≤ 0.05). Similarly, sulfate measured as S and Zn contents were statistically higher in VEG-01B compared to either VEG-01A or VEG-03A (S, *P* ≤ 0.05; Zn, *P* ≤ 0.0001). No significant differences were observed in either Ca, Mg, or Mn content.

**TABLE 7:** Elemental content of lettuce leaf tissue from VEG-01A, VEG-01B, and VEG-03A ground and flight samples.

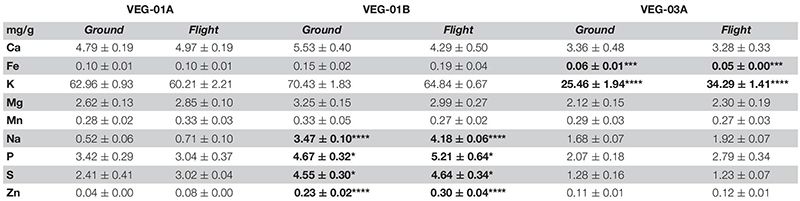

Numbers represent mg/g of dry weight ± Standard Error (*n* = 3). Data highlighted in bold represents elements that are significantly different among all three experiments. Asterisks represent statistical significance calculated from one-way ANOVA followed by Tukey–Kramer test (*P ≤ 0.05, ***P ≤ 0.001, ****P ≤ 0.0001).

### Total Phenolics, Anthocyanins and ORAC Analysis

As a measure of antioxidant capacity, total phenolics, anthocyanins and oxygen radical absorbance capacity (ORAC) were measured on lettuce leaf tissue, flight and ground samples from all three tests (Table 8). Comparing total phenolics, VEG-03A with 15.0 (ground) and 19.6 (flight) mg/g of dry weight (gallic acid equivalents) had the statistically lowest amounts when compared to either VEG-01A or VEG-01B (*P* ≤ 0.001). Anthocyanins measured as cyanidin-3-glucoside equivalents showed no difference among all three experiments compared. Similarly, ORAC units measured among all three experiments did not exhibit any observable variance.

**TABLE 8:** Antioxidant capacity of lettuce leaf tissue from VEG-01A, VEG-01B, and VEG-03A flight and ground samples.

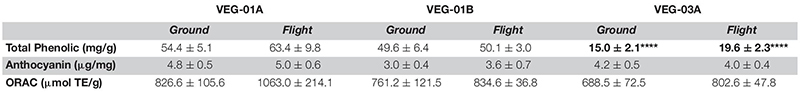

Total phenolic are represented as mg/g of dry weight (gallic acid equivalents), anthocyanins are represented as μg/mg of dry weight (cyanidin-3-glucoside equivalents) and the Oxygen Radical Absorbance Capacity (ORAC) are represented as μmol of trolox equivalents/g of dry weight. Numbers represent unit of dry weight ± Standard Error (*n* = 3). Data highlighted in bold represents values that are significantly different among all three experiments. Asterisks represent statistical significance calculated from one-way ANOVA followed by Tukey-Kramer test (****P-value ≤ 0.0001).

## Discussion

Differences in culturable bacterial and fungal density on the leaves in each of the Veggie plantings can not necessarily be traced to a particular source or environmental condition. However, it is reasonable to conclude that environmental conditions such as temperature and humidity, water levels provided, the microbiota in the air on ISS, and possible human interaction at the time of growth and harvest may contribute to the microbial community and density on the surface of the leaves. Terrestrially, airborne bacteria and fungal spores can land on the surface of leaves and become a part of the epiphytic microbial community albeit transient and dynamic depending on selective pressures (Lindow and Brandl, 2003; Vokou et al., 2012; Maignien et al., 2014). Varying conditions such as humidity, leaf surface moisture and temperature can also effect the colonization and growth of bacteria and fungi on leaf surfaces. There is a positive correlation between fungal sporulation and the duration of leaf surface moisture (Talley et al., 2002; Li et al., 2014). Poor air circulation can cause persistent droplets of moisture on the surface of a leaf due to transpiration producing a micro environment favorable for microbial proliferation. In the case of the three Veggie plantings on the ISS, environmental data collected by HOBO data recorders (Supplementary Table S1) showed that temperature and humidity were slightly higher inside the Veggie chamber during the first 22 days of VEG-03A growth than the other two Veggie tests. Additionally, germinating seedlings in the VEG-03A test had excess moisture in the early period of growth, likely due to a fan malfunction, when compared to the VEG-01A and VEG-01B plants. It was during this experiment that our team identified a consistent anomaly with the Veggie hardware – specifically that when power is cycled to the hardware, upon restarting the fan will read “low” but it will actually be off. This problem also led to issues of excess moisture in an earlier Zinnia test but a repetition of this with lettuce confirmed the anomaly. In contrast, VEG-01A and VEG-01B tests had no fan issues, but instead the plants had some periods of low water early on (see Massa et al., 2017a). Given that Veggie is a manually watered system and that fluid behavior in microgravity differs considerably from that on Earth, these early Veggie experiments involved considerable variation in fluid addition as both the ground teams and the astronauts struggled to effectively water the crops. Logged data from VEG-03A were not collected for the duration of the 64 days experiment so it is speculative to conclude any correlation between microbial counts and Veggie chamber conditions.

Key to this work is determining the microbiological food safety of Veggie grown crops. Bacterial and fungal counts on fresh plant material are not necessarily an indication of the food quality and microbiological safety of the item and vary depending on the type of plant (Leff and Fierer, 2013; Qadri et al., 2015). Studies show that leafy greens grown in the field or greenhouse have a range of approximately 4–8 log_10_ CFU bacterial counts per gram of sample while yeast and mold counts on lettuce are reported in the range of 2–5 log_10_ (Valentin-Bon and Jacobson, 2008; Rastogi et al., 2012; Jackson et al., 2013; Holvoet et al., 2015; Wood et al., 2015; Hagenmair and Baker, 2016; Oyinlola et al., 2017; Fröhling et al., 2018; Zhang et al., 2018). The microbial counts on lettuce leaves grown on ISS in VEG-01A, B and both harvests from VEG-03A were relatively low on average when compared to farm or market produce counts of similar type. In addition, none of the target human pathogens were detected in culture based testing and 16S community sequencing. These results demonstrate that from a microbiological perspective the Outredgeous variety of lettuce grown in Veggie was safe for human consumption.

Microbiological data collected from lettuce and reported in the literature provides evidence that different parts of the plant, i.e., leaves and roots support distinct microbial communities (Ibekwe and Grieve, 2004; Toju et al., 2019). While our culture based isolation of bacteria recovered a few species unique to leaves many of the isolates present on the phyllosphere were also present in the roots. Previous work on the relationship of the phyllosphere bacterial community and airborne bacteria by Vokou et al. (2012) demonstrated that colonization of the phyllosphere is not always related to the aerial microbial community within the plants proximity and may include commensal and symbiotic taxa as well as environmentally indigenous transients. *Staphylococcus* has been isolated on the ISS on both surfaces and in the air (Venkateswaran et al., 2014, 2017) so it is not unlikely that plants in Veggie chambers would be exposed to these types of bacteria. Microbiological surveys done on a variety of surfaces and materials on the ISS reported many of the same bacteria and fungi that we isolated from plant material grown in Veggie. We compared bacterial and fungal isolates from this study of Veggie grown plants with isolates described in a study by Venkateswaran et al. (2017) which characterized the microbiology of ISS surfaces. More than 50% of the genera of bacteria and 100% of the fungi isolated and identified on ISS surfaces were also isolated from Veggie samples.

NASA reported monitoring results of space station samples including air surfaces and water from 1998 to 2012 using cultivation methods (Castro et al., 2004; Yamaguchi et al., 2014). In these studies, the two most commonly isolated genera from water samples collected between 2009 and 2012 were *Burkholderia* sp. and *Ralstonia* sp. These are persistent and common in station potable water as well as terrestrial water sources and have been isolated and identified from Space Shuttle potable water (Koenig and Pierson, 1997; Ryan et al., 2007) so it is not unexpected that these were also isolated in the roots and leaves of both flight and ground control plants.

Plants, regardless of growth conditions, harbor an indigenous population that may affect plant health and, if utilized as a food source, human health. One of the important findings in this study is the similarity and densities in the dominant leaf and root microbial community members as those reported in the literature grown under very different conditions (Dees et al., 2015).

Culturable microbes include approximately 10% of the community microbial participants. Research on core rhizosphere and phyllosphere associated bacterial microbiota (symbiotic and pathogenic) have been described using culture-independent sequencing methods (Panstruga and Kuhn, 2019). Next generation sequencing provides an alternative method to identify non-culturable microorganisms. It is important to identify these organisms as it may provide insight into the safety and health quality of the plants/crops being harvested and consumed. Each of the bacterial and fungal isolates identified in this study were confirmed with sequencing results, however, many additional genera were identified. Research studies indicate Proteobacteria, Bacteroidetes, and Actinobacteria phyla dominate communities, though differences may occur between host plant species at lower taxonomic levels (Panstruga and Kuhn, 2019). Sequencing data from the three Veggie experiments followed these trends.

Lettuce leaf tissue in all three Veggie experiments was dominated with *Thermogemmatispora*, a gram positive, sporulating, soil organism that has been known to produce secondary metabolites provided to plants as a secondary resource (Komaki et al., 2016). *Thermogemmatispora* has been found in lettuce seeds in high abundance but was reduced in root and leaf tissues (unpublished data, Khodadad et al). *Bradyrhizobium* and *Mesorhizobium* are also commonly found in soils as they may participate in nitrogen fixation (Okubo et al., 2012). *Bradyrhizobium* has also been noted in higher abundance in low nutrient soils (Okubo et al., 2012). Both microbes were present in higher abundance in flight root samples and were low or undetectable in leaf tissue with the highest abundance in VEG-03A roots. VEG-03A was grown for longer periods of time and may have depleted the nutrient levels in the substrate. In addition, microbes commonly associated with water were also detected in both leaf and root tissue. These microbes mentioned previously include *Ralstonia*, *Burkholderia, Sphingomonas, Cupriavidus*, and *Pseudomonas* were identified in all leaf and root samples of all three Veggie experiments. These have been identified as present on ISS as well as in the ISS water system (Castro et al., 2004; Checinska et al., 2015; Venkateswaran et al., 2017).

It is important to note that several of the microbes identified may be potential endosymbionts with plants or fungi and be present as epiphytic or endophytic organisms. One example is *Chitinophaga*, found in all flight and ground root samples. Though it is unknown what the function of this bacteria may have been within these Veggie communities, it has been found to interact with fungal species present in plants by enhancing nutrient uptake and growth, altering plant water interactions or deterring potential pathogens (Shaffer et al., 2017). These interactions have the capability of shaping plant health and productivity by accessing and making additional carbon sources available (Kivlin et al., 2011; Shaffer et al., 2017). To determine their role and benefit to plants or fungi would require additional flight and ground studies. As fungi were present in each flight and ground sample community, it is important to note those in abundance and their persistence over time. Fungi have been detected on the ISS in various locations (Venkateswaran et al., 2014, 2017). Leaf and root systems of VEG-01A and B were surveyed for the presence of fungi and compared. *Alternaria, Microidium*, and *Phaeoacremonium* were the top three fungal genera identified with sequencing. *Alternaria*, an Ascomycota, has been described as a saprophyte that may decompose organic matter, and increase in communities with high humidity (Patriarca, 2016). They may also associate with other fungal genera such as *Fusarium* or *Stachybotrys*. Though neither of these fungal genera were identified in VEG-01A, both were first detected in VEG-01B samples with higher incidence in ground samples therefore, they may have been introduced as a contaminant. *Phaeoacremonium* is a plant associated endophyte previously associated with lettuce and woody plants (Mostert et al., 2005) while *Microidium* may be an opportunistic plant pathogen especially in high humidity environments (Braun and Cook, 2012; Meeboon and Takamatsu, 2017).

Plants require macronutrients (N, P, K, Ca, Mg, S) and micronutrients (Fe, B, Mn, Cu, Zn, Mo) for their growth and development (Epstein, 1972). These elements are essential and play critical roles in multiple plant processes. Comparing all three experiments, VEG-01B had higher elemental contents for Na, P, S, and Zn, whereas VEG-03A had the lowest amounts for Fe and K. Considering the chronological order of the experiments onboard the ISS, VEG-01B was grown a year later than VEG-01A, which raises the possibility of a change in the ISS water, but that may not be the only variable as plants grown in microgravity experience stresses from environmental conditions, so it would be impractical to draw any conclusions from these differences. VEG-03A, which was grown 1.5 years after the VEG-01B, was the sequential harvest experiment. As described in section “Materials and Methods,” there were four repeated harvests (38, 45, 59, and 64 days after initiation) of this experiment and only two (day 45 and day 64) were returned to earth. Romaine lettuce in field settings can take 75 days to fully mature, however, controlled environment growth is generally faster. By the final harvest photos indicated that some of the plants seemed to be transitioning to a reproductive growth phase, so it is possible that stresses from space flight may have pushed the plants to initiate senescence and that could provide a possible explanation for slightly lower elemental levels of some elements in VEG-03A samples (Maillard et al., 2015).

Similarly, differences in the levels of total phenolics among VEG-01A, VEG-01B, and VEG-03A samples could be attributed to the environmental conditions. There have been known challenges associated with irrigation of plants in the Veggie hardware and VEG-01 showed evidence of insufficient and excess (in case of zinnia, VEG-01C) water in the root zone (Massa et al., 2017a). Studies have shown that either insufficient watering (drought-like, Sarker and Oba, 2018) or over-watering leading to hypoxia (Rajapakse et al., 2009) can induce production of phenolic compounds and free-radical scavenging activities. Again, there are not enough data to make any conclusions about the effects of the sequential harvesting method on plant ionome or phenolic content and more robust experiments are needed.

Numerous Veggie tests have been conducted on the ISS, the plant growth evaluated, and in some cases the leaves consumed. Three plantings of red romaine lettuce were considered here (VEG-01A, VEG-01B, and VEG-03A). Through culturable and non-culturable methods of microbial analysis, the Veggie tests demonstrated diverse microbial communities with no potential human pathogens detected and therefore could provide a safe supplement to the astronauts’ diet. Chemical analysis provided evidence of significant changes in elemental and antioxidant content which may be an important factor to consider for nutritional value in future, long duration, exploration missions.

## Data Availability Statement

The datasets generated for this study can be found in GeneLab accessions: VEG01A: GLDS-267, VEG01B: GLDS-268, and VEG03A: GLDS-269.

## Author Contributions

GM, RW, MR, and TS contributed to the design of the work with MH, LS, JR, and CK completing research and data analysis. AD contributed to the data analysis. All authors contributed to the development of the manuscript and final version to be published.

## Conflict of Interest

CK, MH, LS, AD, and JR are employed by AECOM Management Services, Inc., under the Laboratory Services and Support Contract at NASA, Kennedy Space Center, Merritt Island, FL, United States. The remaining authors declare that the research was conducted in the absence of any commercial or financial relationships that could be construed as a potential conflict of interest.
