# Supplementary material for: Microbiological and Nutritional Analysis of Lettuce Crops Grown on the International Space Station
Source: Front Plant Sci. 2020 Mar 6;11:199. doi: 10.3389/fpls.2020.00199 (PMC7067979; doi:10.3389/fpls.2020.00199)

Table S1. ISS and Veggie environmental data from May-June 2014 (VEG-01A), July-August 2015 (VEG-01B), and October-November 2016 (VEG-03A). Veggie interior environment was measured using an inserted HOBO data logger (U12-011 Onset, Borne, MA) for VEG-01A and VEG-03A. Data points are averages ± standard deviations. ISS environment data were downlinked from the space station sensors (not immediately adjacent to the hardware) and are averages for the entire experiment. VEG-01 A and B ran for 33 days, while VEG-03A ran for 64 days. HOBO data are averages for the entire 33 days of experimentation from germination until harvest for VEG-01A, and for the first 22 days of the experiment for VEG-03A. Unfortunately the data logger capacity was exceeded at this point as pillows had been stored for several months before initiation. ISS environment data were collected every minute for VEG-01A and every 5 minutes for VEG-01B and VEG-03A, and HOBO data were collected at 15 minute intervals. Number of data points for each row is indicated in the first column. Although the day-to-night transitions were instantaneous, for calculation simplicity to avoid the photoperiod transition period, night data are averages of the hours between midnight and 05:45 AM and day data are averaged between 10:00 AM and 15:45 PM to give 24 data points for each period.


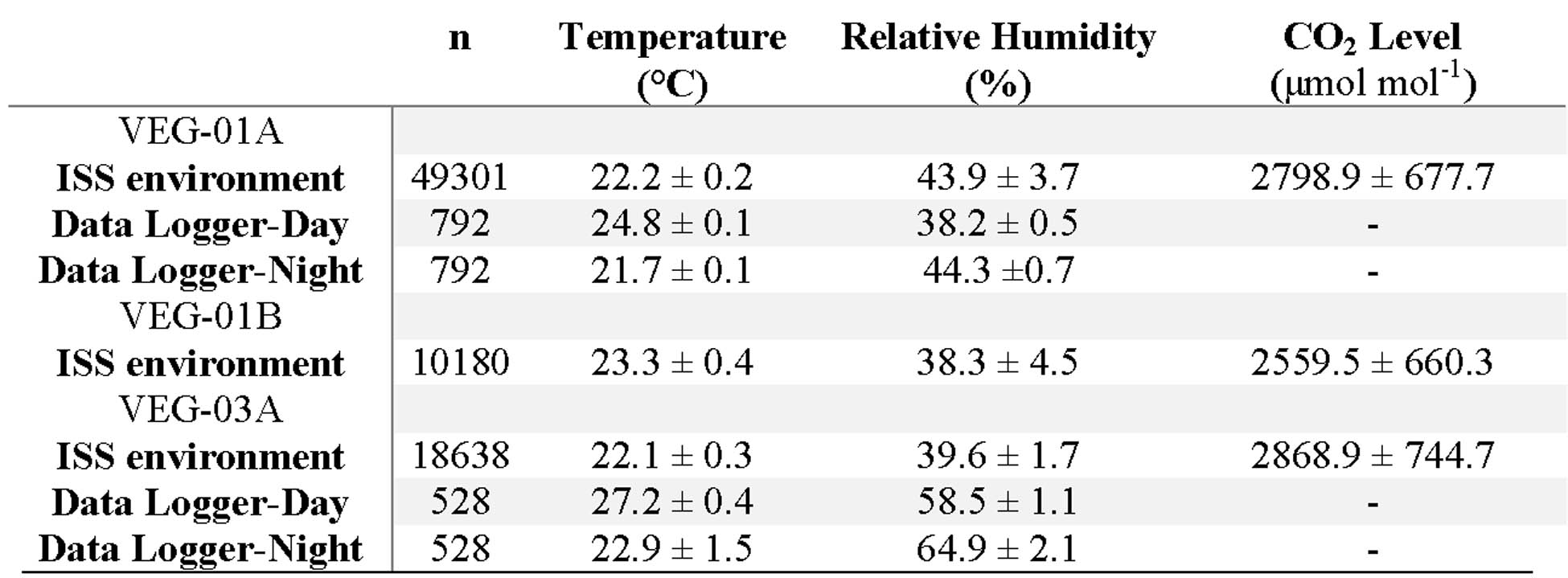

Supplement: Supplementary file 1 [file Table_1.DOCX]
